# A neuropsychological feedback model for memory clinic trainees

**DOI:** 10.1186/s12909-023-04903-z

**Published:** 2024-01-08

**Authors:** Melissa E. Rindge, Lauren Strainge, Maureen K. O’Connor

**Affiliations:** 1 Neuropsychology Service, Bedford VA Healthcare System, 200 Springs Rd, Bedford, MA 01730 USA; 2 Geriatric Research Education and Clinical Center (GRECC), Bedford VA Healthcare System, Bedford, MA USA; 3 Department of Neurology, Boston University, Boston, MA USA

**Keywords:** Neuropsychology, Feedback, Training, Memory clinic, Dementia, Mild cognitive impairment

## Abstract

Memory clinics that specialize in evaluating and treating cognitive decline in older adults are increasingly common and serve as an important training setting for neuropsychology practicum students, interns, and postdoctoral residents. Following a neuropsychological evaluation, trainees are tasked with sharing results, diagnoses, and treatment recommendations, a practice referred to as feedback. Despite the importance and complexity of providing feedback in this setting, no specific model of feedback delivery exists to guide trainees when learning this crucial skill within a memory clinic. The following article presents a feedback model for memory clinic trainees and details its development based on best practices available in the literature. The feedback model aims to promote trainees’ confidence in their clinical skills and increase patient and visit partner understanding of evaluation results. It is also our hope that this model will advance the field of education within neuropsychology.

**Supplementary Information:**

The online version contains supplementary material available at 10.1186/s12909-023-04903-z.

## Background

As the number of older Americans increases [1], specialty memory clinics have become an increasingly important setting for the evaluation and treatment of cognitive concerns in older adults [2]. Memory clinics have also become common training sites for neuropsychology practicum students, interns, and postdoctoral residents, who often play crucial roles in determining if a neurocognitive disorder (i.e., mild cognitive impairment, dementia) is present.

As in other settings, neuropsychology trainees within memory clinics are tasked with sharing evaluation results, diagnoses, and recommendations, a practice referred to as feedback. Though several excellent models and guidelines exist to inform approaches to feedback [3–13], none are specific to memory clinics and few are expressly aimed at educating trainees.

This gap in the literature is surprising, as memory clinics pose several unique clinical challenges (see Table 1). First, it is guaranteed that memory clinic trainees will encounter patients with a level of cognitive impairment that can interfere with their ability to understand their results. Therefore, trainees must be able to deliver and adapt their feedback approach in a way that promotes a patient’s understanding of these results. Second, memory clinic feedback sessions often include visit partners (e.g., spouses, children, caregivers), and novice clinicians must be able to navigate complex relationship dynamics while conveying nuanced information. Third, memory clinic trainees often provide new diagnoses of neurocognitive disorders associated with chronic and terminal illnesses (e.g., Alzheimer’s disease) and must be able to communicate this difficult news with expertise and empathy. This differs from other neuropsychology settings where patients are often already aware of their diagnosis (e.g., epilepsy, head injury) prior to the evaluation.

Providing feedback is a complex new skill for novice clinicians, and the lack of a structured training model has the potential to cause several negative consequences for trainees and their patients (see Table 1). Having no feedback model may increase the likelihood of miscommunication, such as the overuse of medical jargon or trainee avoidance of difficult topics [14]. Trainees may be particularly prone to such pitfalls in a memory clinic when faced with the varied emotions patients and visit partners may experience upon receiving a neurocognitive diagnosis [15–19]. The potential consequences of such shortcomings can be significant. Suboptimal feedback may leave memory clinic patients and visit partners feeling confused, overwhelmed, or unsupported, which can impede their subsequent engagement in healthcare [19–22]. Similarly, trainees may feel inadequate or distressed in response to difficult feedback experiences, which can erode self-efficacy and increase risk for burnout [13, 21, 23].

**Table 1:** Justification for a feedback model for memory clinic trainees

Unique Challenges for Providing Feedback in Memory Clinics	Consequences of No Feedback Model for Trainees
Memory clinic trainees will encounter patients with a level of cognitive impairment that can interfere with their ability to understand results and must be able to deliver and adapt their feedback in a way that promotes a patient’s understanding of these results.	Trainees may miscommunicate with patients and visit partners (e.g., overuse of medical jargon).
Memory clinic trainees often have to navigate complex relationship dynamics between patients and their visit partners while sharing nuanced information to them during feedback.	Patients and visit partners may be left feeling confused, overwhelmed, or unsupported during/after a feedback session.
Memory clinic trainees are often the first to share new diagnoses with patients and visit partners that may be associated with chronic and/or terminal illnesses (e.g., Alzheimer’s disease).	Trainees may feel inadequate or distressed in response to sharing emotionally difficult diagnoses to patients/visit partners, which can reduce trainee feelings of efficacy and increase risk for burnout.

In such a challenging clinical context, common methods of teaching feedback (e.g., observing while a supervisor provides feedback) run the risk of falling short. To this end, a more structured approach for teaching feedback to neuropsychology trainees within a memory clinic would advance both clinical care and neuropsychology education.

## Feedback model development

Given our goal to effectively teach neuropsychology trainees, this model’s development was informed by the initial stages of Kern’s model of curriculum development in medical education [24].

### Problem identification and needs assessment

Our model was developed within a Department of Veterans Affairs (VA) memory clinic embedded in a larger neuropsychology service. This clinic provides neuropsychological evaluations to older adults with memory concerns and serves as a training site for practicum students, interns, and postdoctoral residents. Our combined training/supervising experiences within this clinic and other memory clinics made it clear that there was a need for a structured approach to teaching feedback skills within this setting. However, given that a review of the literature yielded limited information on any such teaching methods, we sought to develop a novel training tool specifically designed for trainees to address this need.

### Initial model development

#### Model goals and objectives

Having identified memory clinic neuropsychology trainees as our intended learners and consumer of this model, we chose to design a step-by-step, patient-centered feedback model. This approach was taken because less-experienced clinicians tend to learn best with structured frameworks [25–28], and trainees may be uncertain about how best to apply more general guidelines or suggestions [13, 29].

Consistent with ethical and training guidelines in neuropsychology, it was paramount that our model promotes high quality clinical care by incorporating all key components of standard neuropsychology feedback ([8–13]; see Table 2). Second, it was necessary that our model be structured, yet flexible enough to address the developmental needs of trainees across different stages of learning [30].

**Table 2:** Standard components of feedback from other models incorporated into the memory clinic feedback model

Source	Setting for Feedback	Components Incorporated into the Memory Clinic Feedback Model
Carone et al. [8]	Sharing invalid test results	1. Describe conclusions based on objective data. 2. Describe test results in the context of comparing the patient’s scores to groups of patients with various degrees of neurological disease. 3. Debrief with the patient about the content and process of the feedback session (e.g., answering questions and addressing concerns).
Carone et al. [9]	Traumatic brain injury	1. Inquire about/address patient anxieties and concerns about the evaluation. 2. Review diagnosis. 3. Discuss prognosis. 4. Discuss factors that can contribute to symptom presentation. 5. Review treatment options.
Carone et al. [10]	Normal neuropsychological test results	1. Explain to the patient that a thorough and comprehensive report has been completed. 2. Review test scores with the patient and explain how scores were compared to individuals in a normative samples with similar education levels. 3. Review recommendations.
Gass & Brown [11]	General neuropsychological practice	1. Review the purpose of testing. 2. Define the tests as behavioral samples that represent important domains of daily functioning. 3. Explain the test results. 4. Describe the patient's strengths and weaknesses. 5. Address diagnosis and prognosis. 6. Make recommendations.
Gorske & Smith [12]	General neuropsychological practice	1. Set the agenda for the feedback session. 2. Discuss the patient’s strengths and weaknesses. 3. Elicit the patient’s reaction to the test results. 4. Link test performance to real-world functioning. 5. Utilize motivational interviewing techniques. 6. Discuss recommendations.
Postal & Armstrong [13]	General neuropsychological practice	1. Reorient the patient and family to the purpose of the evaluation. 2. Gather more information to solidify formulation. 3. Lead with the bottom line. 4. Share the data/results to the patient.

#### Model content and educational strategies

The content and educational strategies incorporated into the current model were drawn from existing models of neuropsychology feedback and the literature on healthcare education strategies, patient-clinician communication, and delivering dementia diagnoses (see Table 3).

**Table 3:** Influences for the memory clinic feedback model

Influences	Elements Incorporated into the Memory Clinic Feedback Model	Source
Existing neuropsychology feedback models	Feedback is considered a standard component of neuropsychological evaluations and serves as a clinical intervention that includes: Sharing a diagnosis, psychoeducation, emotional support, and treatment planning	Carone et al. [8] Carone et al. [9] Carone [10] Connery et al. [6] Gass & Brown [11] Gorske & Smith [12] Postal & Armstrong [13]
Neuropsychology ethical and professional guidelines	1. Psychologists make an effort to explain assessment results to patients. 2. APA’s general ethical principles: Beneficence/Nonmaleficence, Fidelity and Responsibility, Integrity, Justice, Respect for People’s Rights and Dignity. 3. Psychologists maintain awareness of the potential negative impact and outcome of assessment measures on patients. 4. Neuropsychology training competencies: Professionalism, individual and cultural diversity, ethical/legal standards and policy, reflective practice/self-assessment/self-care, relationships, scientific knowledge and methods, and research/evaluation.	APA [3, 4] Nelson et al. [31]
Patient-Clinician Communication Principles	Principles of transparency, full disclosure, and providing accurate and current information to the patient.	Ha & Longnecker [32] Paget et al. [33]
SPIKES-D Model	Address patient emotions, summarize results, and strategize treatment plan.	Peixoto, Diniz, Godeiro [21]

The current framework was strongly influenced by existing models of neuropsychological feedback [8–13] and ethical/professional guidelines for neuropsychologists [3, 4, 31]. Consistent with these guidelines, our model assumes that feedback is a standard component of a neuropsychological evaluation. Feedback in memory clinics serves as a clinical intervention that incorporates psychoeducation, support, and treatment planning and goes beyond simply informing the patient of their diagnosis. Consistent with the practice of many neuropsychologists [34], feedback within this model is intended to occur 2 to 4 weeks after the evaluation, which allows supervisors time to review trainees’ work and gives trainees time to conceptualize the case and plan feedback with their supervisors. This model can also be appropriate for clinics that offer feedback sooner to patients (e.g., same-day feedback), as having a framework ahead of time can quickly orient trainees to the process of feedback and be a resource that supervisors can use at the beginning of the training year and when providing informal or brief supervision.

This feedback model is detailed enough to support trainee learning while also being adaptable to clinician style, patient presentation, and clinical demands [35]. The model’s framework emphasizes the most critical components of feedback to ensure standards for quality care are met and feedback goals are addressed. Consistent with psychotherapy treatment manuals [36–38], sample scripts and timing suggestions are also provided to help trainees manage their time while organizing a large amount of complex information [13, 14]. Though many neuropsychologists provide feedback in-person [8–13], barriers to attending in-person appointments are common among older adults [39, 40], particularly since the COVID-19 pandemic [41]. Given that patients are equally satisfied with telehealth and in-person visits [42, 43], this model is adaptable to in-person and telehealth formats to promote access to care.

Patient-clinician communication principles of transparency, full disclosure, and the provision of accurate and current information are reflected throughout the model [32, 33] and have been shown to improve patient understanding and clinical follow-up [35, 44, 45]. Given that dementia diagnoses are common within memory clinics, the Six-Step Protocol for Delivering Bad News for Dementia (SPIKES-D [21]) was also a key influence, particularly its emphasis on addressing patient emotions, summarizing results, and strategizing a treatment plan.

### Input from neuropsychology colleagues

Following completion of the initial feedback model, we sought input from two neuropsychology colleagues within our medical center who also supervise trainees and teach them how to provide feedback. Both colleagues felt the model was comprehensive and well-constructed and offered several suggestions that were incorporated. These suggestions primarily included clarifying language around diagnostic impressions and recommendations (e.g., identifying use of clinical jargon, providing examples of how to discuss the cognitive impacts of mood, sleep, and other treatable conditions) that were incorporated into the final model. 

## The memory clinic feedback model

The core sections of the feedback model are summarized below. See Additional file 1: Appendix A for the model in its entirety. Though this model veers on the side of comprehensive for the sake of education and training, we also offer suggestions when working with patients whose cognitive impairment is suspected to impact their ability to understand evaluation results. In these cases, trainees should be briefer and more direct.

Regardless of the patient’s cognitive ability, trainees should always treat the patient as the primary consumer during the evaluation and feedback session when using this model. For instance, trainees should focus on the patient during the discussion (i.e., asking them directly if they have any questions, making regular eye contact) and not talk about the patient with visit partners as if the patient were not in the room. When patients with cognitive impairment present to the evaluation alone, it is recommended that trainees strongly encourage them to bring a visit partner to the feedback session. If they are unable to do so, trainees should make a concerted effort to involve the referring provider and/or get permission from the patient to involve social supports (e.g., family members, caseworkers) to assist the patient in following through on recommendations. A brief and easily understandable summary of evaluation results and recommendations should be provided to all patients, but particularly for patients who have difficulty comprehending their results. A summary can both serve as a reminder for the patient but can also be shared with a support person to help the patient follow through on recommendations.

This model can also be applicable to trainees who do not conduct their own testing (i.e., with use of a psychometrist or another trainee in tiered supervision) with just a few minor adjustments to the sample. For instance, the trainee could identify the name of the individual who did the testing while making it clear that they and their supervisor interpreted the results. Because there may be less opportunity for a trainee to develop rapport with the patient if they do not do their own testing, they may wish to spend additional time reorienting the patient and visit partner to their role and the purpose of the visit at the beginning of the feedback session.

### I. Introduction to the feedback session

Trainees begin with a brief introduction to the feedback session to re-establish rapport, manage patient expectations, and remind patients/visit partners of the goals for the appointment. Presenting an agenda can alleviate anxiety for patients who are unsure what to expect, promote engagement, and provide an opportunity for patients to consent or assent (in the event a patient lacks capacity) to feedback [21, 35]. Trainees should also provide reassurances around concerns that might otherwise interfere with the patient’s ability to fully engage in the session (e.g., fears that results may show they are “crazy” or “need to be locked up” [9, 12]).

For patients suspected to have difficulty comprehending their results, it is recommended that trainees be brief and explicitly state the reason for the referral (e.g., “I had an appointment with you a few weeks ago to see if you have dementia”).

### II. Review purpose of the evaluation and relevant history

Patients with memory problems often require reminders about the evaluation itself. Review of the referral question, presenting concerns, and factors pertinent to the diagnosis serve as a reminder and can act as a touchstone for points made later in feedback. This overview also helps patients/visit partners know their concerns have been incorporated into the provider’s impressions and provides an opportunity for patients/visit partners to correct inaccuracies or share information that has arisen since the evaluation [13].

For patients suspected to have difficulty comprehending their results, it is recommended that trainees only review one or two of the most significant concerns, symptoms, or aspects of their history. For instance, a patient being diagnosed with Alzheimer’s disease may be reminded that this evaluation was done because there was concern that he/she has wandered outside of the home and gotten lost in their neighborhood. If there was a medical event associated with the onset of cognitive dysfunction, the trainee may choose to focus on this (e.g., “It sounds like there have been a lot of changes since your stroke a year ago”).

### III. Describe how test results are interpreted

Patients and visit partners may not automatically consider neuropsychological testing to be a valid reflection of real-world abilities [13, 14]. Brief education about test interpretation can demonstrate clinician credibility and communicate that the patient’s individual circumstances have been taken into account [10, 11]. For instance, a patient concerned that testing is not sensitive to their cognitive decline because they “always had an excellent memory” may be reassured to know that test interpretation takes premorbid functioning into account. Results may also be more impactful when patients/visit partners understand that scores are interpreted using age-related norms and deficits cannot be explained as “just getting older.” For patients suspected to have difficulty comprehending their results, it is recommended that trainees keep this section brief and just remind patients that tests to measure memory (or other cognitive domains of concern) were given.

### IV. Share the test results

Individuals with cognitive problems may be easily overwhelmed by new information, so trainees first provide patients/visit partners with a summary statement about test results [13]. Trainees then transition to a more detailed discussion of strengths and weaknesses across cognitive domains in patient-friendly language. Descriptions of individual scores are not recommended [8, 11], and results should be discussed in the context of the patient’s presenting concerns with real-world examples. It is also important to discuss areas of preserved functioning, which can help reassure patients/visit partners that they can continue to engage in meaningful activities [11–13].

The overall summary of test results is repeated to reiterate the primary findings and reduce the risk of the patient, visit partner, or trainee getting “lost in the weeds” [13, 14]. Before proceeding, trainees see if patient/visit partners have questions with open-ended prompts (e.g., “What questions can I answer?”), which are more likely to elicit responses from patients/visit partners compared to close-ended questions.

For patients suspected to have difficulty comprehending their results, it is recommended that trainees still provide a “take home” message about the results, emphasizing that the patient had more difficulty than expected given their age and background. However, trainees should also focus on discussing test performance based on the main area of cognitive concern (e.g., “On testing, you had a lot more difficulty with memory than I would expect for your age and background” for a patient with suspected Alzheimer’s disease).

### V. Provide diagnostic impressions

Most patients and visit partners prefer direct communication about their diagnosis [16, 32, 46]. However, the nuances of diagnoses common in memory clinics can be difficult to navigate. This model ensures that trainees provide education about diagnostic criteria for neurocognitive disorders, framed in the context of the patient’s cognitive concerns, test results, and daily functioning. The model also highlights the importance of explicitly stating the patient’s diagnosis to promote clear communication and reduce clinician avoidance of difficult conversations (e.g., explicitly sharing a diagnosis of dementia or a neurodegenerative disease). For patients suspected to have difficulty comprehending their results, it is recommended that the diagnosis be discussed in combination with etiology (discussed below).

### VI. Discuss etiology

For patients with objective cognitive impairment, it is useful to clarify that neurocognitive diagnoses (e.g., dementia) are broad terms that can be caused by numerous conditions. Many patients/visit partners are understandably unaware of the clinical distinctions between dementia and Alzheimer’s disease and may be confused if terms are not explained.

Because most memory clinic patients/visit partners find diagnostic labels helpful [47], this model emphasizes the importance of identifying all potential etiologies and their prognosis, beginning with the primary etiological consideration and its associated prognosis. For instance, if Alzheimer’s disease is considered the most likely etiology, that fact that the patient’s cognition is expected to worsen over time is stated directly.

Complex clinical presentations are common in memory clinics and pose a challenge to clear communication of results. If the etiology of a patient’s cognitive impairment is multifactorial, this is also stated directly and each potential etiology is explained. For cases where the etiology is unclear, trainees explain this uncertainty, describe possible etiologies, and clarify why a definitive diagnosis cannot be made. For patients who do not meet criteria for a neurocognitive disorder, trainees validate the patient's cognitive concerns and provide education about potential contributing factors (e.g., sleep disturbance). If specific etiological concerns are not relevant (e.g., remote history of mild traumatic brain injury), this is also addressed directly.

Throughout this discussion, trainees utilize clinical skills to ensure that the questions and emotional needs of the patient/visit partners are addressed [35]. Psychotherapeutic interventions (e.g., empathic listening, grief counseling, suicide risk assessment) can be integrated as needed. The model requires trainees to prompt for questions and concerns at the end of this phase in order to reduce the likelihood of inadvertently rushing through or avoiding emotionally difficult topics. It is also helpful at this stage to briefly assess the patient/visit partner’s understanding of the results, diagnosis, and etiology to address any misperceptions.

For patients suspected to have difficulty comprehending their results, it is recommended that trainees combine sections V and VI and provide a “take away” message by explicitly stating the diagnosis and/or etiology (e.g., “I’m concerned you have Alzheimer’s disease”). These patients would likely still benefit from a brief discussion about the definition of dementia/major neurocognitive disorder (i.e., a decline in cognitive abilities impacting daily activities) and how certain conditions (e.g., Alzheimer’s disease, vascular disease) can cause dementia.

### VII. Provide recommendations and assist with treatment planning

Feedback is intended to aid treatment planning and support the patient’s well-being [11–13]. Therefore, all recommendations are discussed with the patient/visit partner and are explained in the context of the evaluation results [13, 34]. When recommending referrals to other services, the clinician seeks the patient/visit partner’s buy-in and engages in motivational interviewing and goal setting as necessary. Emotional support around challenging treatment recommendations (e.g., assisted living, advance directive planning) is also provided.

For patients suspected to have difficulty comprehending their results, it is recommended that trainees focus on only one or two of the most pertinent recommendations (e.g., neuroimaging, seeing a neurologist).

### VIII. Conclude

Feedback can be a highly emotional experience for patients/visit partners [15–19]. It is important for trainees to recognize that patients/visit partners may feel overwhelmed and need time to process the information. Reminding patients that information from feedback is also available in a written report [10] or in a summary letter is often reassuring and reduces the burden on the patient/visit partner to remember everything.

For patients suspected to have difficulty comprehending their results, it is recommended that trainees provide another take away message about the diagnosis/etiology and most pertinent recommendation(s).

## Discussion

Though many neuropsychological feedback models exist [8–13], there are no models specifically designed for trainees to help them learn this complex skill within memory clinics. Our model addresses this gap by incorporating existing feedback frameworks, ethical/professional guidelines, and available literature on healthcare education strategies, patient-clinician communication, and delivering dementia diagnoses. We also believe that this model has utility for novice supervisors (e.g., clinicians new to supervising, senior-level trainees providing tiered supervision) so that they have a framework to teach supervisees how to provide feedback.

There is no one-size-fits-all approach to feedback [8]. The work of memory clinic trainees is complex, and this model will not apply to every patient, situation, or clinic demands. Furthermore, trainees and their supervisors may have different opinions as to what and how much information should be shared during feedback and may find that not all the steps within this model are applicable to their work. Though a careful review of the literature was incorporated into this model, there are sure to be many other effective methods for teaching and providing feedback. Despite these limitations, this feedback model can serve as a starting point for trainees to improve patient and visit partner understanding of the neuropsychological evaluation and enhance their own clinical skills and confidence.

### Limitations and future directions

Though there is a clear gap in the literature regarding educational tools for teaching feedback to memory clinic trainees, we did not conduct a formal needs assessment (e.g., trainee survey, focus group) prior to developing this model. It is, therefore, possible that our model may not address all the needs of neuropsychology memory clinic trainees. Given the specificity of our VA setting, it is also possible that the model may not generalize perfectly to all memory clinics.

For next steps, investigating trainee perceptions of the model and its impact on trainee learning/performance will undoubtedly contribute to iterative improvement of the current feedback model. Additional research to evaluate patient and visit partner responses to feedback delivered by trainees using this tool (e.g., understanding of results, perceived rapport, engagement with follow-up) will also be critical.

### Conclusions

Despite these limitations, our feedback model in its current form can serve as a valuable tool for neuropsychology trainees who provide the complex intervention of feedback to memory clinic patients. Although evaluating the efficacy of this feedback model will be important moving forward, it incorporates current best practices and addresses an important gap in the neuropsychology education literature. The availability of such a tool for memory clinic trainees is of particular importance, as they are likely to encounter the increasing number of older adults seen within this setting in their future stages of training and career.

### Supplementary Information

**Additional file 1.**

## Data Availability

Not applicable.

## Abbreviations

VA: Department of Veterans Affairs
SPIKES-D: Six-Step Protocol for Delivering Bad News for Dementia
