# Supplementary material for: A neuropsychological feedback model for memory clinic trainees
Source: BMC Med Educ. 2024 Jan 8;24:40. doi: 10.1186/s12909-023-04903-z (PMC10773085; doi:10.1186/s12909-023-04903-z)
Supplement: Supplementary file 1 — Additional file 1. [file 12909_2023_4903_MOESM1_ESM.pdf]

The Memory Clinic Feedback Model

2023

Melissa E. Rindge[1], Lauren Strainge[1], & Maureen K. O'Connor[1,2,3]

[1]Neuropsychology Service, Bedford VA Healthcare System
Bedford, MA

[2]Geriatrics Education and Clinical Center (GRECC), Bedford VA Healthcare System
Bedford, MA

[3]Department of Neurology, Boston University
Boston, MA

I. Introduction to the Feedback Session [1-2 min.]

Share the purpose of feedback, provide an agenda, and emphasize that patients/visit partners can ask questions at any time.

Example: *We're here today to discuss the results of your neuropsychological testing from [date], to share our recommendations for things we believe may be helpful for you, and to give you (and your [visit partner]) a chance to ask any questions. Our primary goal today is to make sure that you feel you understand everything we've talked about, so please feel free to chime in at any time with questions, thoughts, or concerns. I'm going to start by briefly reviewing what you told me when we met. This is your opportunity to correct me if I got anything wrong or to add anything new. Then we'll discuss the results of the evaluation and our recommendations and have time for questions. Does that sound like a good plan for today? [Field any initial questions].*

II. Review Purpose of the Evaluation and Relevant History [3-5 min.]

Provide an overview of the referral source and question, presenting concerns, relevant history, and any factors pertinent to the diagnosis.

Example: *So just to recap, you were referred to us by [referring provider] because you (and [visit partner]) expressed concerns that your memory has been getting worse over the past few years. You told me about trouble remembering conversations and losing things, getting lost when driving, and having more trouble remembering to take your medications. You also have some health conditions, like diabetes and high blood pressure, which you've been having a harder time managing. You also told me that you've been having trouble sleeping because you're worried about your memory. Do you feel like that's a good summary? Has anything come up since the last time we met that you think would be important for me to know about?*

III. Describe How Test Results are Interpreted [2-3 min.]

Provide brief psychoeducation about the way results are assessed to make it clear to the patient/visit partner(s) that the evaluation results have taken their individual history into account.

Example: *When we completed testing, I asked you to do a lot of different tasks to measure different kinds of memory and thinking. One reason I spend so much time getting to know you at the beginning is so I can compare how you did on the tests to how I would expect you to do, before any of these changes started. That is, given what you told me about yourself and how old you are, are you performing the way I would expect, or are there some areas where you having more difficulty? Then I look to see if there are patterns of difficulties across your test scores. This helps me understand what might be causing the difficulties you (and [visit partner]) have been noticing.*

IV. Share the Test Results [5-10 min.]

A. Provide a summary at both the start and end of section to emphasize the take-away message.

Examples:

-Overall, you did well on most of the tests I gave you.

-You did well on some tests, but there were areas where you had significant difficulty.

-The testing was hard for you, and showed that you're having more difficulty than I would expect.

B. Discuss areas of strength and weaknesses.

- **Tie this back to specific tests as necessary, particularly if the patient noted difficulties on certain tasks.**
- **Connect this information back to their reported symptoms.**

Example: *On the tests, there were some areas of thinking where you did as well I would expect based on your age. These included your ability to process visual information and memory. In these areas, you're still doing just as well as I would expect you to do.*

In other areas, though, you had more difficulty than I would expect based on your age and background. You had quite a bit of difficulty on the language tests we did. You even told me that you were having a hard time when I asked you to name some pictures of objects. This fits in with the difficulty you told me you've been having coming up with words. The tests support that and show me that you're having a much harder time with language than I would expect for someone your age.

C. Prompt for questions/reactions before moving on.

Example: *What questions do you have about what I've said so far?*

<u>IV. Provide Diagnostic Impressions</u> [3-5 min.]

A. Provide education about what is required for diagnosis of a major/mild neurocognitive disorder.

- **Tie this back to examples of the patient's reported functioning.**
- **Clearly state the diagnosis listed in the report and reiterate the rationale based on the criteria.**
- **If no neurocognitive disorder is diagnosed, state this clearly.**

Example for Major Neurocognitive Disorder/Dementia: *Based on the test results, you're having more difficulty with memory than you used to. You (and [visit partner]) told me that these difficulties are impacting your ability to do daily tasks as well as you used to. You're having more difficulty keeping track of when you have taken your medications and your bill payments.*

When people are having problems with their thinking that we can measure on our tests, like you are, and those problems are interfering in their ability to perform daily tasks, we say that person has dementia. Dementia means those two things: that you are having problems with thinking and these problems are causing difficulties with your ability to do day-to-day tasks in the way you always have.

Dementia can also be called Major Neurocognitive Disorder and the two mean the same thing.

Example for a Mild Neurocognitive Disorder/Mild Cognitive Impairment: *Based on the test results, you're having more difficulty with attention, multitasking, and staying on task than you used to. However, you (and [visit partner]) told us that you're still able to go about your day-to-day activities without much difficulty. You are still able to cook for yourself and keep track of your appointments and when you've taken medication.*

When people are having problems with their thinking that we can measure on our tests, like you are, but those problems do not interfere with their ability to perform daily tasks, we say that person has mild cognitive impairment. 'Cognitive impairment' because you're having problems with thinking, and 'mild' because those problems aren't really impacting your ability to go about your day-to-day activities as well as you ever have. This is the term that I used in my report and is the formal diagnosis we are giving you: mild cognitive impairment.

Mild cognitive impairment can also be called Mild Neurocognitive Disorder and the two mean the same thing.

Example for no neurocognitive diagnosis:

Based on the test results, you don't meet criteria for a formal neurocognitive disorder, which means that when I tested your thinking, I don't see any areas that are concerning for cognitive impairment.

I know you feel that you're struggling more with memory in your daily life, though, and we'll talk about some things that I think may be contributing to that. [Move into etiology discussion.]

B. Transition to discussion of etiology

Example: *The next question is why are you having these difficulties?*

<u>VI. Discuss Etiology</u> [10-15 min.]

A. Explain that Major Neurocognitive Disorder/Dementia and Mild Neurocognitive Disorder/Mild Cognitive Impairment are umbrella terms and can be caused by many different things.

- **If no neurocognitive disorder is present, explain that many factors can impact thinking.**

> **Example:** *There are many things that can cause [dementia/mild cognitive impairment/thinking difficulties] and this label alone does not tell us what is causing the problem, sort of how knowing that someone has a headache doesn't tell us what is causing the problem, because lots of different things can cause headaches, like stress, head injuries, and so on.*

B. Discuss etiological considerations, beginning with the primary concern.

- **Be direct in stating the name and prognosis associated with each.**
- **Identify the specific pattern of results that suggests the primary etiology (e.g., rapid forgetting) so the patient and visit partner(s) understand what led to your conclusions.**

B1. If the etiology is multifactorial, state so directly and describe each consideration, beginning with the most relevant.

B2. If the etiology is unclear, state so directly. Describe possible factors and explain why a more definitive diagnosis cannot be provided. This should lead directly into discussion of recommendations (i.e., to aid in differential diagnosis).

B3. If no neurocognitive disorder is present, discuss factors that could be contributing to subjective complaints (e.g., normal aging, psychiatric distress, sleep/pain, psychosocial factors). Include all etiological considerations presented in the report. Provide brief education related to how each etiological factor impacts cognition/the brain.

B4. If specific etiological concerns are not relevant (e.g., a remote mild head injury is non-contributory, a patient is worried about Alzheimer's disease but their profile is not concerning), state so directly and provide any necessary education around their concerns.

Examples for Primary Etiology:
- Lots of things can cause dementia/thinking problems. Based on the pattern of your test results, I think the most likely cause of your problems is Alzheimer's disease.

-Lots of things can cause dementia/thinking problems. Based on the pattern of your test results and some new symptoms you reported, I am concerned about the possibility of Dementia with Lewy Bodies.

Example for Unclear Etiology: *Lots of things can cause dementia/thinking problems. Based on the pattern of your test results, it is not entirely clear to me what may be causing these difficulties, though some of your health conditions like poorly controlled diabetes may be a factor.*

Example for Secondary Etiologies: *In addition to your history of stroke, there are other factors going on in your life that I think are making it harder for you to think, learn, and remember. These include anxiety and difficulty sleeping. While I think these things are making life more difficult for you, I believe the biggest factor impacting your thinking right now is the stroke you had.*

C. Prompt for questions/reactions before moving on.

Example: *What questions can I answer? How does what I've said so far fit in with your concerns and what you've been noticing at home?*

D. Once patient questions have been addressed, check for understanding. Address any inaccuracies, misperceptions, or inability to explain the key points before going on.

> **Example:** *We've discussed a lot of information, so I want to check that I've been able to explain things clearly. In your own words, can you tell me what we've covered so far about the results of testing and what is causing your thinking problems? [Clarify any misconceptions].*

VII. Provide Recommendations and Assist with Treatment Planning [10-20 min.]

Share the recommendations listed in the report.

- **For those referrals to other services, clearly elicit whether or not the patient/visit partner would like a referral to be placed.**
- **Tie recommendations back to the patient/visit partner's concerns and/or test results wherever possible.**
- **Field questions and prompt for reactions/understanding throughout.**
- **Employ motivational interviewing techniques and goal setting as needed.**

> **Example:** *So now that we know what's likely causing your cognitive problems, what can we do about this? Based on everything you (and [visit partner]) told me, and the pattern of the test results, I have a list of recommendations for things that I think may be helpful for you (and [visit partner]). My first recommendation is _____.*

<u>VIII. Conclude</u> [2-5 min.]

Acknowledge that a lot of information has been shared and let the patient/visit partner know that they can reach out for any questions.

**Example:** *I know we covered a lot of material today. Based on what we discussed, I'll place referrals for ______. I will also notify [referring provider] that my report is finished so they can review this information as well. If any additional questions or concerns come up, please don't hesitate to call. You can reach me at [phone number].*
